# Disparities Associated with Decision to Undergo Oncologic Surgery: A Prospective Mixed-Methods Analysis

**DOI:** 10.1245/s10434-024-15610-4

**Published:** 2024-06-13

**Authors:** Robert M. Van Haren, Melinda Butsch Kovacic, Aaron M. Delman, Catherine G. Pratt, Azante Griffith, Lana Arbili, Krysten Harvey, Eshika Kohli, Ahna Pai, Alique Topalian, Shesh N. Rai, Shimul A. Shah, John Kues

**Affiliations:** 1 University of Cincinnati Cancer Center, University of Cincinnati College of Medicine, Cincinnati, OH USA; 2 Department of Rehabilitation, Exercise, and Nutrition Sciences, University of Cincinnati College of Allied Health Sciences, Cincinnati, OH USA; 3 Department of Pediatrics, Cincinnati Children’s Hospital Medical Center, University of Cincinnati College of Medicine, Cincinnati, OH USA

**Keywords:** Modifiable provider-based barriers, Patient-physician relationships, Complex surgical malignancies, Cancer disparities, Decision-making strategies

## Abstract

**Background:**

Underrepresented minority patients with surgical malignancies experience disparities in outcomes. The impact of provider-based factors, including communication, trust, and cultural competency, on outcomes is not well understood. This study examines modifiable provider-based barriers to care experienced by patients with surgical malignancies.

**Methods:**

A parallel, prospective, mixed-methods study enrolled patients with lung or gastrointestinal malignancies undergoing surgical consultation. Surveys assessed patients’ social needs and patient-physician relationship. Semi-structured interviews ascertained patient experiences and were iteratively analyzed, identifying key themes.

**Results:**

The cohort included 24 patients (age 62 years; 63% White and 38% Black/African American). The most common cancers were lung (*n* = 18, 75%) and gastroesophageal (*n* = 3, 13%). Survey results indicated that food insecurity (*n* = 5, 21%), lack of reliable transportation (*n* = 4, 17%), and housing instability (*n* = 2, 8%) were common. Lack of trust in their physician (*n* = 3, 13%) and their physician’s treatment recommendation (*n* = 3, 13%) were identified. Patients reported a lack of empathy (*n* = 3, 13%), lack of cultural competence (*n* = 3, 13%), and inadequate communication (*n* = 2, 8%) from physicians. Qualitative analysis identified five major themes regarding the decision to undergo surgery: communication, trust, health literacy, patient fears, and decision-making strategies. Five patients (21%) declined the recommended surgery and were more likely Black (100% vs. 21%), lower income (100% vs. 16%), and reported poor patient-physician relationship (40% vs. 5%; all *p* < 0.05).

**Conclusions:**

Factors associated with declining recommended cancer surgery were underrepresented minority race and poor patient-physician relationships. Interventions are needed to improve these barriers to care and racial disparities.

**Supplementary Information:**

The online version contains supplementary material available at 10.1245/s10434-024-15610-4.

In 2003, the Institute of Medicine released the landmark report ‘Unequal Treatment: Confronting Racial and Ethnic Disparities in Health Care’, which detailed multiple ways race and ethnicity remain significant predictors of poor quality of health care received after accounting for socioeconomic status.^1^ Surgical disparities have been described,^2-4^ and adult minority patients with hepatic, pancreatic, biliary, lung and esophageal malignancies (collectively, complex surgical malignancies) have a higher incidence of disease and worse overall survival than non-Hispanic White patients.^5-12^ The factors that contribute to complex surgical malignancy disparities in patients from underrepresented groups span the continuum of care and can be classified into five distinct categories: patient-based, provider-based, system and access, clinical care and quality, postoperative care, and rehabilitation.^3,13^ The majority of cancer disparities research focuses on *patient-based* (race, ethnicity, socioeconomic status, medical comorbidities, behavior) and *system-based* (insurance status, institutional/hospital-level policies, health care access, cost) factors that contribute to worse outcomes,^9,14-16^ likely due to the relative ease of determining associations between race/ethnicity and outcomes in large, national, de-identified databases. However, little is known regarding the *provider-based* (patient-physician interaction, including rapport, trust, communication, empathy, unconscious bias, cultural competency, language use, and awareness of health disparities) factors that contribute to worse outcomes in minority patients with complex surgical malignancies.^3^

Currently, there is a lack of literature to suggest how underrepresented minority patients experience their providers, whether their provider experience facilitates or presents barriers to care, or what they would label as their most significant barrier to equitable care.

The goal of this project was to identify modifiable provider-based factors that impact the receipt of equitable care for underrepresented minority patients with complex malignancy utilizing a patient-centered, mixed-methods approach. Moving forward, we can develop future intervention trials that can potentially mitigate these barriers that influence cancer survival. This is impactful in its focus on the patient and allows patients lived experiences to guide the search and solution for modifiable barriers to care among underrepresented cancer patients.

## Methods

A prospective mixed-methods study was performed and patients with lung or gastrointestinal malignancies undergoing surgical consultation were enrolled from June 2021–August 2022. This study was conducted at the University of Cincinnati Cancer Center and was approved by the Institutional Review Board (IRB; #2021-0394). Written informed consent was obtained from all participants.

The target population was patients with a newly diagnosed hepatic, pancreatic, biliary, or thoracic malignancy who were scheduled for outpatient surgical consultation with thoracic or surgical oncology. Some patients did not have pathologic confirmation of their malignancy at the initial clinic visit. Inclusion criteria were patients aged 18 years and older and fluent in English, while the exclusion criteria included recurrent cancer and patients who had already started treatment for their new cancer diagnosis.

Demographic information, oncologic details, treatment received, and survival were obtained from the electronic medical records. Patients were asked to complete two surveys (Electronic supplementary Figs. 1 and 2) and a semi-structured interview. The surveys included the Accountable Health Communities Health-Related Social Needs Screening Tool created by the Centers for Medicare and Medicaid Services.^17^ This tool screens for social determinants of health: living situation, food, transportation, utilities, safety, financial strain, employment, family and community support, education, physical activity, substance use, mental health, and disabilities. The second survey instrument was created by the investigators to focus on patients’ individual cancer-specific diagnosis and assessed the degree to which patients experienced provider-based (unconscious bias, cultural competency, language use, clinical competency, motivation and awareness of health disparities, supportive hospital policies) and systematic barriers to care (health care access, insurance status, institutional/hospital-level policies, cost containment strategies, time to diagnosis, time to appointment with surgeon, time until surgery). Five-point Likert scales were used to quantify the significance of each individual barrier to care that the patient experienced.

After the surveys were completed, a semi-structured interview was conducted by trained research personnel. The initial interview guide was constructed using themes found in the literature on patients’ perceptions and experiences with healthcare providers during the diagnosis and treatment of cancer. A preliminary conceptual framework was developed to classify the types of barriers to equitable care experienced by underrepresented minority patients, with a particular focus on provider-based barriers. Semi-structured interviews focused on patients’ experiences with their new diagnosis, their interactions with healthcare providers, and their overall experiences with the healthcare system. The interview was constructed using open-ended questions and was refined through a process of constant comparison as new themes and concepts arose from interviews.

In some cases, caregivers who had accompanied the patient to their visit were present during interviews. Although the interview was conducted with the patients, there were occasions when the caregiver provided input. All interviews were audio-recorded with permission, transcribed verbatim, and evaluated shortly after completion to continuously update the interview guide and to assess new major concepts and ideas being introduced.

The transcripts were de-identified and the investigators analyzing the transcripts were not involved in conducting the interviews. The transcripts were analyzed using an iterative thematic assessment. Initial themes were developed by three investigators (RVH, AP, JK) through the independent review of two transcripts. The initial themes were tested on two additional transcripts, and modifications were made based on this review. All transcripts were subjected to review and re-review by two investigators (EK and JK).

Descriptive statistics are reported using the mean and standard deviations (SDs) for continuous variables, and frequencies and percentages for categorical variables. Comparisons between different groups were compared using the Chi-square test and independent t-test. Data collected from the interviews were analyzed using qualitative content analysis.

## Results

In total, 24 patients consented to participate in this study. The mean (SD) age was 62 (12) years. Of these participants, 67% (*n* = 16) were male, 63% (*n* = 15) were White, and 38% (*n* = 9) identified as Black race. The majority of patients were single (*n* = 12, 52%), six were married (26%), two were widowed (9%), and three were divorced (13%). The most common cancers were lung (*n* = 18, 75%) and gastroesophageal (*n* = 3, 13%).

Food insecurity (*n* = 5, 21%), lack of reliable transportation (*n* = 4, 17%), and housing instability (*n* = 2, 8%) were common among participants (Table 1). Thirty-eight percent of participants (*n* = 9) reported difficulty in paying for very basics such as food, housing, medical care, and heating. Participants reported a lack of trust in their physician (*n* = 3, 13%) and their physician’s treatment recommendation (*n* = 3, 13%), as well as a perceived lack of empathy (*n* = 3, 13%), lack of cultural competence (*n* = 3, 13%), and inadequate communication (*n* = 2, 8%) from their physician (Table 2). Additionally, 29% of patients reported that their race/ethnicity played a role in how they were treated as a patient.

**Table 1:** Accountable health communities health-related social needs screening tool

	No. (%)
Housing instability	2 (8)
Food instability	5 (21)
Lack of reliable transportation	4 (17)
Utility needs	0 (0)
Interpersonal safety	4 (17)
Difficult to pay for very basics such as food, housing, medical care, and heating	9 (38)

**Table 2:** Patient-physician relationship survey

	No. (%)
Physician did not spend enough time discussing diagnosis and treatment options	2 (8)
Physician did not address questions and concerns	3 (13)
Physician did not understand patient’s goals of care	3 (13)
Physician did not participate in shared decision making	2 (8)
Physician race/ethnicity played a role in how patient was treated	3 (13)
Patient race/ethnicity played a role in how patient was treated	7 (29)
Lack of empathy	3 (13)
Inadequate communication	2 (8)
Lack of cultural competence	3 (13)
Lack of trust in physician	3 (13)
Lack of trust in treatment recommendation	3 (13)

**Fig. 1:**
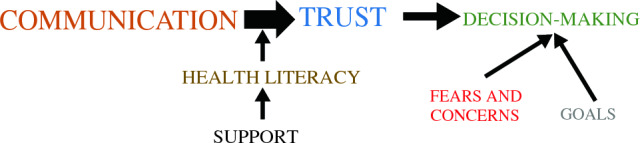
Qualitative model of themes

Qualitative analysis identified eight themes, each of which had several subthemes. Five of the themes were determined to be major themes and three were determined to be minor themes, based on the frequency with which they appeared in the interviews. Table 3 outlines each of the themes and subthemes. There were positive and negative comments about all of the themes, but the positive comments were a clear majority. The major themes regarding decision to undergo surgery, i.e. communication, trust, health literacy, fears, and decision making, were organized into a qualitative model (Fig. 1).

**Table 3:** Qualitative themes and subthemes

Theme	Subtheme
*Major themes*	
Communication	Ease of communication
	Quality of interactions
	Understandability
	Caregiver expressed clear advocacy
Trust	Professional
	Cares about patient
	Wants the same things as the patient
	Educated
	Care coordination
	Past experiences with medicine and UC Health
	Doctor-patient relationship
Fears and concerns	Procedural fears
	Diagnostic fears
	Loss of independence
	Recovery (time/limitations/etc.)
	Death/prognosis
Health literacy	Understands diagnosis
	Describes procedures
	Linked tests to diagnosis
	Understands treatment/options
	Understands roles of health team members
	Knowledgeable advocate
Decision making	Follow doctor’s advice
	Given options to consider
	Asks questions
	Patient engagement/taking charge
*Minor themes*	
Systems issues	Insurance
	Appointments
	Transportation/other external factors
Complaints	MyChart
	Provider not interested in ‘other issues’ (e.g., home, comorbid conditions, fears)
Support	Family
	Primary care provider
	Others

The final coding rubric included eight themes, each of which had several subthemes. Five of the themes were major themes (communication, trust, fears and concerns, health literacy, and decision making) and three were minor themes (systems issues, complaints, and support), determined by the frequency with which they appeared in the interviews (Table 4).

**Table 4:** Representative quotes for each theme

Theme	Illustrative quotations
Communication	“He talks to you like you’re on the same level” “… I just feel like both of them are easy to talk to … they act like they’re a normal person and not better than anybody else” “Just the way [the doctor] explained to me what’s going on and the steps it’s going to take to fully diagnose what’s going on … she did a great job.” “… and I’ve always been able to call [the doctor] and ask him anything that I had questions about, and if he didn’t have the answer, he called me back with an answer” “I believe they weren’t listening to my concerns” “… sometimes they expect you to just take their word … some of them might say well, this is what we are going to do …”
Trust	“… he seems very competent and knowledgeable …” “… he’s seen a lot over the years and I think he just wants me to be healthy” “But the fact that he seems very caring and compassionate, and confident, gives me a good feeling about having him as my surgeon” “… I’ll go to them once, if I don’t care for him … I don’t go back” “… part of it has to do with the fact that I can’t stand a doctor that’s just arrogant”
Fears and concerns	“I was scared. I wasn’t sick, I was scared” “I’ve never been operated on. Yeah, I’m scared” “You know that’s the worst part [to] know that you got something like this wrong with you. I’ve never been shocked like this before” “… if it is going to have me come out of it worse than I when I went it” “My biggest concern is I’d like to be able to wake up and walk and talk and move around or do something”
Health literacy	“And they noticed from one of my scans that I had some nodules in the upper part of my right lung. This led to a more definitive scan … which revealed the presence of cancer cells in my upper right lobe of my lung” “… sometimes I know what they are talking about, like the day I just found out that they had to remove a part of the lung”
Decision making	“I agree with the (decision my doctor) made … I agree with that completely” “I don’t use emotions very much to make decisions. I use facts and all the facts were fine” “I got enough information where I can relay it with my wife and we can discuss it together”
Systems issues	“… we then see if my insurance company will pay for it. You know, I don’t like bills cutting into my income if I had to pay something, like I did get a recent bill that was unexpected” “… the explanation (for why we have to go different places) is that, at least for the oncologists, they have a separation … They treat breast cancer in Westchester, and they treat other cancers here. … we did ask initially, you know, could we go to West Chester … because it’s closer?” “… now it's … three separate hospitals, because my physician is through (Hospital A), the lung specialist is through (Hospital B) and then obviously, I'm here for the surgeon, which sometimes can make it kind of difficult”
Complaints	“As far as you know, divorces and what not … you know, the abuse itself, I want them to be aware of it. I’d like for them to get involved”
Support	“So I don't have time to sit around and be depressed and lonely because I have so much love around me, so much support. Yeah, I'm good” “… there's going to be a barrier that comes along, but I've got pretty good family support too. So it's, it definitely makes it easier”

### Major Themes

*Communication:* The most common theme discussed by patients in regard to their oncologic surgeon was communication. Patients perceiving their communication with providers to be positive tended to comment on how comfortable they felt talking with their physician, and felt heard. Several patients also commented on their doctor’s responsiveness to questions and general availability. Although most comments about communication were positive, the dissatisfaction that some patients perceived was typically related to believing that the physicians were not listening to them or being too paternalistic.

*Trust:* Patient perceptions of their provider’s trustworthiness was generally connected to the quality of communication. The most common factors related to patients’ trust in their provider were the fact that they appeared very competent, and they displayed empathy and concern for the patient. The patients who expressed trust issues often had previous negative experiences with doctors and the healthcare system. Their lack of trust was not always directed at their current physicians, but they were more skeptical than patients who had positive or more neutral past experiences.

Several patients commented that their doctors had been recommended to them by others and they had a reputation for being very good. Several patients also noted that they felt their current circumstances (i.e. a diagnosis of lung cancer) put them in a position where they had to trust the surgeons and care team. This was especially true when the patient had little prior experience with the healthcare system.

*Fears and Concerns:* The third major theme patients expressed was fear regarding their new diagnosis, treatment, and long-term outcomes. For many patients this is the first time they have had a potentially life-threatening diagnosis. Several patients expressed concerns about permanent disabilities and difficult recovery. This included a loss of independence and increased burden on family members.

*Health Literacy:* Many parts of the interviews revealed differing levels of health literacy among the participants. Patients with a better understanding of their condition and navigating the healthcare system reported better communication with their providers and higher levels of trust. Several patients noted that they had a family member or friend who was a healthcare provider helping them understand and navigate their current situation. The general indicators of higher health literacy were the use of current terms for tests, understanding the link between tests and diagnosis, and correctly explaining tests results and treatment options.

Lower health literacy was indicated by a general lack of knowledge of tests, procedures, and links between test findings, diagnoses, and treatment options. Patients with indications of lower health literacy also tended to trust their providers because they felt providers were educated and competent. Additionally, in comparison with patients displaying higher levels of health literacy, patients exhibiting lower levels tended to ask fewer questions of their providers.

*Decision Making:* Patients were asked to describe next steps in their care and how they were making their decisions. The interviews were conducted early in their care planning, and final decisions about their treatment plan had not been determined. In general, patients tended to take one of three approaches to their treatment: (1) following the advice of their providers; (2) weighing options after gathering information from providers and other sources; and (3) making decisions with their families. In all cases, the opinions of the patients’ doctors were considered.

### Minor Themes

The three minor themes (systems issues, complaints, and support) were mentioned much less often than the five major themes. Minor themes reflected issues that were indirectly impacting patients’ care but were causing concern for the patient and their families/caregivers. For many patients, getting to appointments was challenging because they relied on public transportation or caregivers. Some patients had obligations (i.e., work or family) that limited their availability for appointments. Issues such as parking and appointments in varying locations created barriers to care. Issues with insurance were very rare at this point in the patients care. Most comments related to support describe a spouse or other family members who were available to drive the patients to appointments and participate in the decision-making process. Occasionally, patients mentioned they did not want to be a burden to their family. Several patients had family members who had training as healthcare providers and helped them understand their diagnosis and treatment. These family members also helped patients navigate the complexities of the healthcare system.

Five patients (21%) declined recommended surgery for their malignancy; two patients received treatment with chemotherapy and/or radiation and three patients did not receive treatment for their cancer. Patients declining recommended surgery were more likely to be Black (100% vs. 21%), with lower income (100% vs. 16%), and reported a poor patient-physician relationship (40% vs. 5%; all *p* < 0.05) (Table 5).

**Table 5:** Comparison between patients who received or declined cancer surgery

Variables	Declined cancer surgery [*n* = 5] (%)	Received cancer surgery [*n* = 19] (%)	*p* value
Sex, male	100	58	0.08
Race, Black	100	21	< 0.01
Income, 0–9, 875$	100	16	0.01
Housing instability	20	5	0.29
Food instability	20	21	0.96
Lack of reliable transportation	80	84	0.82
Lack of trust in physician	40	5	0.04
Lack of physician empathy	40	5	0.04
Lack of physician cultural competence	40	5	0.04

In general, the percentage of comments in each of the eight themes were very similar between patients who elected to have surgery and those who did not. Comments about communication and trust were the most predominant for all patients. Over half (52%) of the comments by surgical patients and one-third (33%) of the comments by non-surgical patients were related to these two themes. The differences in the percentage of comments in each theme between surgical and non-surgical patients was 5% or less, with the notable exception of comments about trust. Patients who had surgery mentioned trust in 31% of their comments. Conversely, patients who declined surgery mentioned trust in only 17% of their comments. This difference of 14% was almost three times as large as differences in any other thematic area. Additionally, there were no comments that reflected a lack of trust by patients who underwent surgery. Several comments by patients who declined surgery reflected low levels of trust.

## Discussion

In this study, we found that social barriers to cancer care were common, including food insecurity, lack of reliable transportation, and housing instability. Qualitative analysis identified several major themes for cancer care, such as communication, trust, and decision making (Fig. 1). We observed a high rate (21%) of declining recommended surgery for cancer, and patients who declined surgery were more likely to be Black males, with lower income, and reported a poor patient-physician relationship.

The majority of patients in this study were newly diagnosed with lung cancer, and underrepresented minority patients (African American, Hispanic, multiracial, American Indian) experienced a higher incidence of lung cancer and worse overall survival than non-Hispanic White patients.^12,18^ Our study is consistent with previous research attributing disparate outcomes to differences in socioeconomic status, stage at diagnosis, patient preferences, structural racism, and rates of surgical resection.^12,19,20^ Social determinants of health such as poverty are associated with increased complications and mortality after lung cancer resection, and worse oncologic outcomes.^21^ Our study identified that many patients were experiencing social needs, most notably related to food insecurity, lack of reliable transportation, and housing instability. These findings highlight understudied factors of cancer care that may contribute to racial disparities in cancer outcomes.

We found that most patients reported good or excellent patient-physician relationships, however a small number of patients reported poor relationships with lack of trust in their physician and their physician’s treatment recommendation. In addition to this distrust, patients reported feeling that the physicians displayed a lack of empathy, lack of cultural competence, and inadequate communication. Previous studies have demonstrated similar results and found that a strong patient-physician relationship improves communication, medical decision making, and treatment adherence, and decreases fears.^22^ Other studies have shown that Black patients with lung cancer have less trust in their physicians compared with White patients.^23^ Improving communication and the patient-physician relationship is possible with training^24^ and could help reduce disparities.

Despite attempts to mitigate disparities in lung cancer survival, underrepresented minority patients remain less likely to receive surgical resection, leading to significantly decreased survival.^12,25-27^ One striking example of racial disparities in undergoing surgery was found in the National Lung Screening Trial, a randomized clinical trial with low-dose computerized tomography versus chest radiography, which observed significant racial disparities in the receipt of surgery between Black (65%) and White (93%) males.^28^ Moreover, recent literature has identified ‘patient refusal’ as a reason for decreased receipt of surgical resection among minority patients.^28,29^ In these cases, patients are offered surgical resection, but the patient decides not to undergo surgical resection. Our study also identified a very high rate (21%) of declining recommended oncologic surgery.

Strategies for improving effective communication, increasing trust, and developing care plans that are sensitive to the social and cultural experiences of patients can be addressed through educational interventions and coordination across the healthcare system. Our study provides a foundation to build needed culturally competent education and systematic interventions that can address provider-based barriers to better care for underrepresented minority patients and marginalized populations.

There are existing interventions at local, regional, and national levels, such as free transportation with Road to Recovery by the American Cancer Society, and food banks are offered by many cancer centers. However, one challenge that remains is identifying patients with needs and easily connecting them with these services. Our future work will expand on these findings by developing interventions based on the qualitative model identified in this study. Our future planned interventions, both unique interventions to address health care disparities, include (1) cultural humility and team science training for the clinical team; and (2) enhancing the integrated supportive services team with patient navigation to address barriers to care. This team will also include community ‘lay’ navigators. While individual and team-based training in cultural sensitivity have been utilized to improve cultural humility and practitioner attitudes, this project suggests changes in care coordination can improve survival outcomes for underrepresented patients with cancer.

The present study has several limitations. It was performed at a single cancer center and may have limited generalizability to other patient populations. Most patients had lung cancer and, in a similar manner, these results may not be generalizable to other types of cancer. We decided to include patients with gastrointestinal malignancy because we believe that the social needs and patient-physician relationship barriers to care are not unique to cancer type. In a small number of interviews, caregivers were involved in addition to the patient, and the caregiver responses were not analyzed separately. Our survey’s sample size was small, which could limit the reliability of our quantitative results. Our research personnel were trained in semi-structured interviewing, however there could still be interview bias in our methods. Also, we only included English-speaking patients, which may have influenced our results.

## Conclusions

This investigation identified high rates of surgical decline among underrepresented minorities. Patients who declined surgery were more likely to have poor patient-physician relationships, expressed as a lack of trust, empathy, and cultural competence of their provider. The identification of these provider-based barriers to care related to the patient-physician relationship, and cultural sensitivity provides a solid foundation to develop scalable interventions to mitigate disparities in cancer survival for underrepresented patients. Interventions are needed to improve patient-physician relationships and provide supportive services to patients to help reduce cancer disparities.

### Supplementary Information

Below is the link to the electronic supplementary material.

Supplementary file1 (PDF 302 KB)

Supplementary file2 (DOC 68 KB)
