# Supplementary material for: Disparities Associated with Decision to Undergo Oncologic Surgery: A Prospective Mixed-Methods Analysis
Source: Ann Surg Oncol. 2024 Jun 13;31(9):5757–64. doi: 10.1245/s10434-024-15610-4 (PMC11300547; doi:10.1245/s10434-024-15610-4)
Supplement: Supplementary file 2 — Supplementary file2 (DOC 68 KB) [file 10434_2024_15610_MOESM2_ESM.doc]

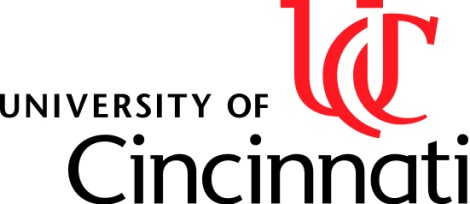
 **College of Medicine**

**Department of Surgery**

University of Cincinnati Medical Center

231 Albert B. Sabin Way

P.O. Box 670558

Cincinnati, OH 45267-0558

Phone: (513) 558-5333

Fax: (513) 558-2585

**Survey 2:**

Do you feel like your doctor spent enough time with you to discuss your diagnosis and treatment options?

1 = strongly disagree

2 = disagree

3 = neutral

4 = agree

5 = strongly agree

Do you feel like your doctor addressed your questions and concerns?

1 = strongly disagree

2 = disagree

3 = neutral

4 = agree

5 = strongly agree

Do you trust your doctor’s recommendation?

1 = strongly disagree

2 = disagree

3 = neutral

4 = agree

5 = strongly agree

Do you believe your doctor was empathetic with your condition?

1 = strongly disagree

2 = disagree

3 = neutral

4 = agree

5 = strongly agree

Do you believe your doctor‘s communication skills were adequate to help you make a treatment decision?

1 = strongly disagree

2 = disagree

3 = neutral

4 = agree

5 = strongly agree

Did you feel like you and your doctor were speaking the same language?

1 = strongly disagree

2 = disagree

3 = neutral

4 = agree

5 = strongly agree

Do you feel like you understand all the necessary work-up needed prior to your therapy?

1 = strongly disagree

2 = disagree

3 = neutral

4 = agree

5 = strongly agree

Do you think you plan on getting the necessary work-up?

1 = strongly disagree

2 = disagree

3 = neutral

4 = agree

5 = strongly agree

Do you trust your doctor?

1 = strongly disagree

2 = disagree

3 = neutral

4 = agree

5 = strongly agree

Did you feel safe with your doctor’s recommendations?

1 = strongly disagree

2 = disagree

3 = neutral

4 = agree

5 = strongly agree

Would you describe your doctor as culturally competent?

1 = strongly disagree

2 = disagree

3 = neutral

4 = agree

5 = strongly agree

Does your doctor understand your goals of care?

1 = strongly disagree

2 = disagree

3 = neutral

4 = agree

5 = strongly agree

Does your doctor participate in shared decision making with you?

1 = strongly disagree

2 = disagree

3 = neutral

4 = agree

5 = strongly agree

Do you think your doctor’s race/ethnicity played a role in how they treated you?

1 = strongly disagree

2 = disagree

3 = neutral

4 = agree

5 = strongly agree

Do you think your race / ethnicity played a role in how you were treated?

1 = strongly disagree

2 = disagree

3 = neutral

4 = agree

5 = strongly agree
